# Resmetirom: An Orally Administered, Smallmolecule, Liver-directed, β-selective THR Agonist for the Treatment of Non-alcoholic Fatty Liver Disease and Non-alcoholic Steatohepatitis

**DOI:** 10.17925/EE.2023.19.1.60

**Published:** 2023-05-01

**Authors:** Gres Karim, Meena B Bansal

**Affiliations:** 1. Department of Medicine, Mount Sinai Israel, New York, NY, USA; 2. Division of Liver Diseases, Department of Medicine, Icahn School of Medicine at Mount Sinai, New York, NY, USA

**Keywords:** resmetirom, nonalcoholic steatohepatitis, hepatic steatosis, hepatic steatohepatitis, liver fibrosis, liver stiffness, nonalcoholic fatty liver disease, THR-β agonists

## Abstract

Non-alcoholic fatty liver disease (NAFLD) encompasses a spectrum of fatty liver disease, including non-alcoholic fatty liver (NAFL) and its more progressive form, non-alcoholic steatohepatitis (NASH). The prevalence of NAFLD/NASH along with type 2 diabetes and obesity is rising worldwide. In those who develop NASH, unlike those with bland steatosis (NAFL), lipotoxic lipids drive hepatocyte injury, inflammation and stellate cell activation leading to progressive accumulation of collagen or fibrosis, ultimately leading to cirrhosis and increased risk of hepatocellular carcinoma. Hypothyroidism is associated with NAFLD/NASH; specifically, intrahepatic hypothyroidism drives lipotoxicty in preclinical models. Agonists of thyroid hormone receptor (THR)-β, which is primarily found in the liver, can promote lipophagy, mitochondrial biogenesis and mitophagy, stimulating increased hepatic fatty acid β-oxidation, and thereby decreasing the burden of lipotoxic lipids, while promoting low-density lipoprotein (LDL) uptake and favourable effects on lipid profiles. A number of THR-β agonists are currently being investigated for NASH. This review focuses on resmetirom, an orally administered, once-daily, small-molecule, liver-directed, ß-selective THR agonist, as it is furthest along in development. Data from completed clincal studies outlined in this review demonstrate that resmetirom is effective in reducing hepatic fat content as measured by magnetic resonance imaging-derived proton density fat fraction, reduces liver enzymes, improves non-i nvasive markers of liver fibrogenesis and decreases liver stiffness, while eliciting a favourable cardiovascular profile with a reduction in serum lipids, including LDL cholesterol. Topline phase III biopsy data showed resolution of NASH and/or fibrosis improvement after 52 weeks of treatment, with more detailed peer-reviewed findings anticipated in order to certify these findings. Longer term clinical outcomes from both MAESTRO-NASH and MAESTRO-NASH OUTCOMES will be a pivotal juncture in the drug’s road towards being approved as a NASH therapeutic.

Non-alcoholic fatty liver disease (NAFLD) encompasses a spectrum of fatty liver diseases, including non-alcoholic fatty liver (NAFL) and nonalcoholic steatohepatitis (NASH).^1^ NAFLD is associated with metabolic disorders, including obesity, hypertension, dyslipidaemia, type 2 diabetes mellitus (T2DM), hypothyroidism and metabolic syndrome.^2^ The diagnosis of NAFLD requires ≥5% histologic or imaging evidence of hepatic steatosis, with exclusion of secondary causes of hepatic steatosis, such as significant alcohol consumption, hereditary disorders and steatogenic medications.^1^ NASH, the more severe and progressive form of the disease, is characterized by inflammation and hepatocyte injury (ballooning) with or without hepatic fibrosis.^1^

Our understanding of the natural history of NAFLD and NASH continues to evolve. In a seminal paired liver biopsy study with a mean follow-up of 13.7 years, Ekstedt et al. conducted a biopsy study found that 16% of patients with NAFLD showed improvement in fibrosis, 43% remained stable, 41% showed fibrosis progression and 5% developed cirrhosis-related complications, thus providing relevant insight into the natural history of NASH with no intervention.^3^ NASH is a heterogenous disease, so multiple factors, including genetic determinants, environmental factors and comorbidities, interact to promote fibrosis progression in specific individuals. Ultimately, fibrosis is the most important predictor of clinical outcomes^4^ and thus represents an important endpoint in clinical trials, while longer-term follow-up can be done to assess clinical outcomes.

The global prevalence of NAFLD is estimated to be 25%, with the highest prevalence in the Middle East and South America and the lowest in Africa.^5^ It is thought to affect 55.5% of individuals with T2DM^6^ and 70–80% of patients with obesity worldwide.^7^ The global prevalence of NASH is about 5%, and among those with T2DM, the prevalence is estimated at 37.3%^6^ and 33.5% among individuals who are overweight or obese.^8^ In USANorth America, the prevalence of NAFLD is estimated to be about 24.13% in the adult population, while NASH is estimated to affect 5–6% of adults.^5,9,10^ Based on data from the Scientific Registry of Transplant Recipients (2002-2019), NASH was the leading cause of liver transplantation in women and the second most common indication overall in waitlisted transplant candidates without hepatocellular carcinoma in the USA.^11^ Markov modelling suggests that by 2030, approximately 100.9 million people will have NAFLD, and 27.0 million people will have NASH with varying stages of fibrosis.^9^ The latter represents a 63% increase in NASH cases from 2015. Of those with NASH, 14 million are projected to have a fibrosis score of F2 or higher (i.e. moderate-to-high fibrosis), which may represent the eligible population for treatment should a drug be approved for therapy.^9^ Despite the increasing prevalence of NASH with the increase in obesity,^10^ no treatment has been approved by the European Medicines Agency or the US Food and Drug Administration (FDA).

## Lipotoxicity as a key driver in the pathogenesis of non-alcoholic steatohepatitis

The pathogenesis of hepatic steatosis is extremely complex but is thought to be conceptionally driven by the excess delivery of free fatty acids to the liver, coupled with increased *de novo* lipogenesis fuelled with excess carbohydrates, particularly fructose.^12,13^ The two major fates of fatty acids in hepatocytes are mitochondrial β-oxidation and re-esterification to form triglycerides.^13^ Triglycerides can be exported into the blood as very-l ow-density lipoproteins or stored in lipid droplets.^14^ Lipid-droplet triglyceride undergoes regulated lipolysis to release fatty acids back into the hepatocyte free fatty acid pool.^14^ When the disposal of fatty acids through β-oxidation or the formation of triglyceride is overwhelmed, fatty acids can contribute to the formation of lipotoxic species that lead to endoplasmic reticulum stress, oxidant stress and inflammasome activation.^14^ Insulin resistance and hyperinsulinaemia further fuel lipid accumulation by not only diverting glucose from glycogen synthesis to *de novo* lipogenesis in the liver but also impacting peripheral lipolytic pathways.^13^

**Table 1:** Histologic stages of hepatic fibrosis

Fibrosis stage	Pathologic features	Implications
F0	No fibrosis	
F1	Portal fibrosis without septa	
F2	Portal fibrosis with few septa	Considered significant fibrosis and baseline inclusion criteria for most NASH clinical trials
F3	Bridging septa between central and portal veins	Advanced fibrosis
F4	Cirrhosis	

*Histologic scoring of fibrosis (F0–F4). Those with score >F2 are considered to have significant fibrosis and constitute the eligible population for most clinical trials for NASH therapeutics*

*NASH = non-alcoholic steatohepatitis.*

When the hepatocytes' ability to handle this stress is overwhelmed, hepatocyte injury and apoptosis result in the recruitment and activation of inflammatory cells and the activation of hepatic stellate cells, which are the predominant cellular source of collagen/fibrosis in response to any chronic liver injury.^15^ The activation of these repair pathways moves diagnosis from simple steatosis (NAFL) to the the more active progressive form of NAFLD, steatohepatitis (NASH).^15^

In its quiescent state, the hepatic stellate cell is vitamin rich and produces predominantly type IV collagen. With injury, it undergoes phenotypic changes, including increased proliferation and contractility and a shift towards producing type I and III collagens, which are characteristic of the cirrhotic liver.^16^ Progressive fibrosis is often defined histologically on a scale ranging from F0 to F4, as shown in *Table 1*.^15^ As fibrosis is the most important predictor of clinical outcomes,^17,18^ improvement in fibrosis has become the most important surrogate endpoint in NASH clinical trials while awaiting longer-term follow-up for clinical outcomes.

**Figure 1:**
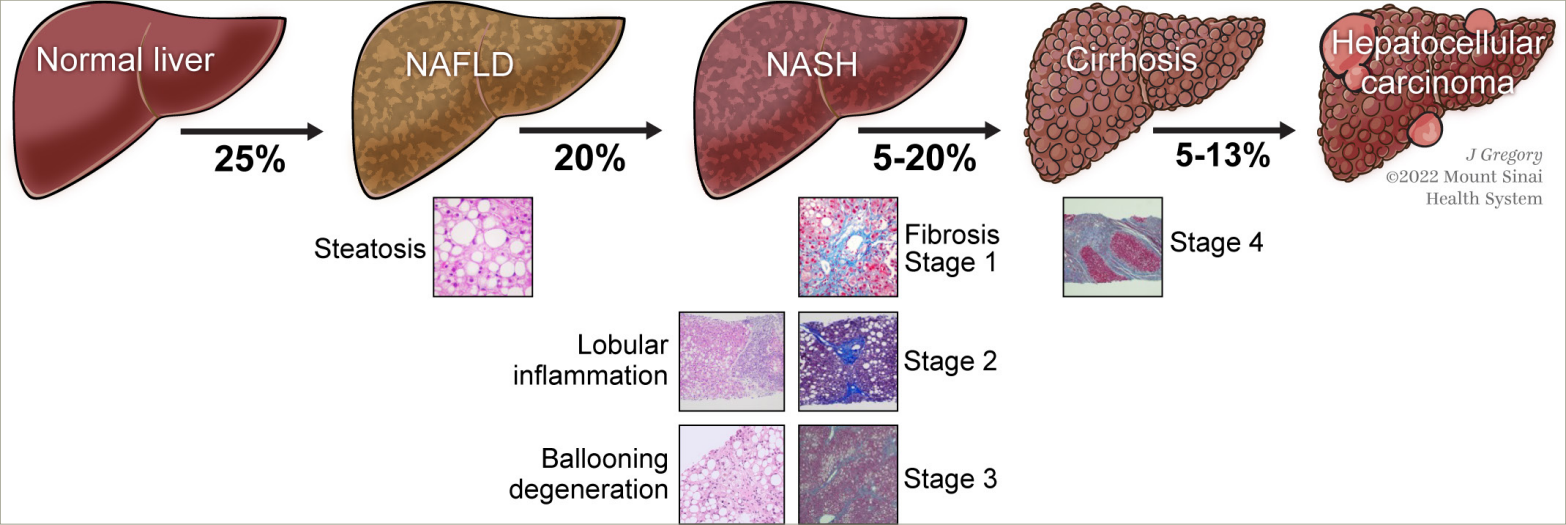
Schematic of the progression of non-alcoholic fatty liver disease to cirrhosis and hepatocellular carcinoma

In the USA, approximately 20% of patients with NAFLD have disease progression to NASH, and up to 20% of patients with NASH may develop cirrhosis within their lifetime (*Figure 1*).^19^ While the most common cause of death in patients with NASH is cardiovascular disease,^20^ the risk of liver-related mortality increases exponentially with fibrosis stage increase,^17^ and liver fibrosis is the most important predictor of liver-related mortality in patients with NAFLD.^18^ Patients with advanced fibrosis are at an increased risk of morbidity and mortality due to the disease progressing to liver failure, cirrhosis and hepatocellular carcinoma.^15,17,18^ In addition to obesity and T2DM, other conditions associated with NAFLD include dyslipidaemia, polycystic ovary syndrome, metabolic syndrome, obstructive sleep apnoea and endocrine disorders including hypogonadism, hypopituitarism and hypothyroidism.^1^ This review will focus on the role of thyroid hormone signalling in NASH pathogenesis and the potential role of thyroid hormone receptor (THR)-β agonists as a treatment strategy.

*About 25% of the population has NAFLD. Of those with NAFLD, 20% progress to NASH. About 5-20% of those with NASH progress to cirrhosis. Hepatocellular carcinoma may result in 5-13% of patients with cirrhosis secondary to NASH.*

*Used with permission from ©Mount Sinai HealthSystem.*

*NAFLD = non-alcoholic fatty liver disease; NASH = non-alcoholic steatohepatitis.*

## Thyroid hormone-mediated regulation of lipid metabolism and mitochondrial activity in the context of non-alcoholic fatty liver disease/non-alcoholic steatohepatitis

### Low serum thyroid hormone levels are associated with non-alcoholic fatty liver disease

The hypothalamus produces thyroid-releasing hormone, which stimulates the production of thyroid-stimulating hormone (TSH) from the pituitary gland.^21^ TSH promotes the secretion of prohormone thyroxine (T4) and triiodothyronine (T3) from the thyroid gland. T3 binds to the THR-α (mainly in the heart and bone) and -β (mainly in the liver) to regulate gene transcription. Population studies have shown associations between NAFLD and overt hypothyroidism, subclinical hypothyroidism and low thyroid hormone in the normal range.^22–25^ Patients with higher baseline TSH levels and no evidence of liver disease were more likely to develop NAFLD.^26^ In addition, the prevalence of NAFLD was found to significantly increase as serum TSH level increased, even after adjusting for age, gender and smoking status.^23^ Moreover, a recent systematic review and meta-analysis by Mantovani et al. found hypothyroidism to be significantly associated with the presence and severity of NAFLD.^27^ Given the association between hypothyroidism and NAFLD, Bruinstroop et al. treated with T4 20 Asian men who have normal thyroid gland function with T2DM, euthyroidism, hepatic steatosis on ultrasound and alanine aminotransferase levels less than three times the upper limit of normalT4; dosing was titrated to achieve TSH levels 0.34-1.70 mIU/L, as the lowest prevalence of NAFLD was seen in those with TSH<1.70 mIU/L.^28^ Patients were then treated with a maintenance dose of thyroxine for 16 weeks. Reduction in hepatic steatosis was assessed using magnetic resonance imaging-derived proton density fat fraction (MRI-PDFF). Overall, a 12% decrease in intrahepatic lipid content was observed, with a 23% reduction in those over the age of 50 years. However, in patients with subclinical hypothyroidism or euthyroidism, the beneficial effects of longer treatment with thyroid hormone would need to be weighed against long-term clinical adverse effects, such as atrial tachycardia, arrhythmias and loss of bone density through the effect of THR-α. As THR-β is the predominant isoform in the liver, specific targeting may be a better approach.

### The effect of thyroid hormones on autophagy, mitophagy, mitochondrial biogenesis and β-oxidation

Autophagy is a normal physiologic metabolic process wherein a cell consumes its own redundant or damaged organelles to fuel regeneration of newer healthier organelles. T3 induces autophagy in the hepatoma cell line HepG2 in a dose-dependent manner and specifically promotes lipophagy, resulting in the delivery of additional internal free fatty acids to the mitochondria for β-oxidation.^29^ In addition, T3 promotes mitochondrial oxidation due to a combination of mitophagy and mitochondrial biogenesis.^30^ Taken together, these results show that thyroid hormones can regulate lipid homeostasis via autophagy and help to explain how thyroid hormones increase oxidative metabolism through effects on mitochondrial turnover and activity.

### Intrahepatic hypothyroidism as a driver of nonalcoholic steatohepatitis

In a mouse NASH model where mice were fed a Western diet with fructose for 16 weeks, increased steatosis, inflammation and fibrosis were associated with a statistically significant decrease in intrahepatic T4 and T3.^31^ In addition, in a dietary mouse model of NASH, administration of thyroid hormones decreased hepatic triglyceride content (3.19 ± 0.68 mM/g liver versus 8.04 ± 0.42 mM/g liver) and hydroxyproline (1.44 ± 0.07 mg/g liver versus 2.58 ± 0.30 mg/g liver) compared with mice with untreated NASH.^32^ Moreover, thyroid hormones restored autophagy and mitochondrial biogenesis to increase β-oxidation of fatty acids and reduced lipotoxicity, oxidative stress, hepatic inflammation and fibrosis.^32^

In the liver, deiodinase type 1 (DIO1) converts the prohormone T4 to the bioactive hormone T3. Interestingly, in a model of early NASH, DIO1 levels and activity are increased suggesting that there is a compensatory increase to handle excess lipids.^31^ However, low DIO1 levels and activity have been observed in humans and rodents with advanced NASH,^33^ and DIO1 knockdown leads to increased intrahepatic lipid content.^31^

At the cellular level, in both rodent and human healthy livers, hepatocytes strongly expressed DIO1 and stromal cells weakly expressed DIO3.^33^ Very little DIO1 was detected in other non-parenchymal cells of the liver. During injury, hepatocyte expression of DIO1 decreased, whereas stromal expression of DIO3 increased, particularly in myofibroblasts.^33^ In patients, this was also reflected by increased serum reverse T3 (rT3).

**Figure 2:**
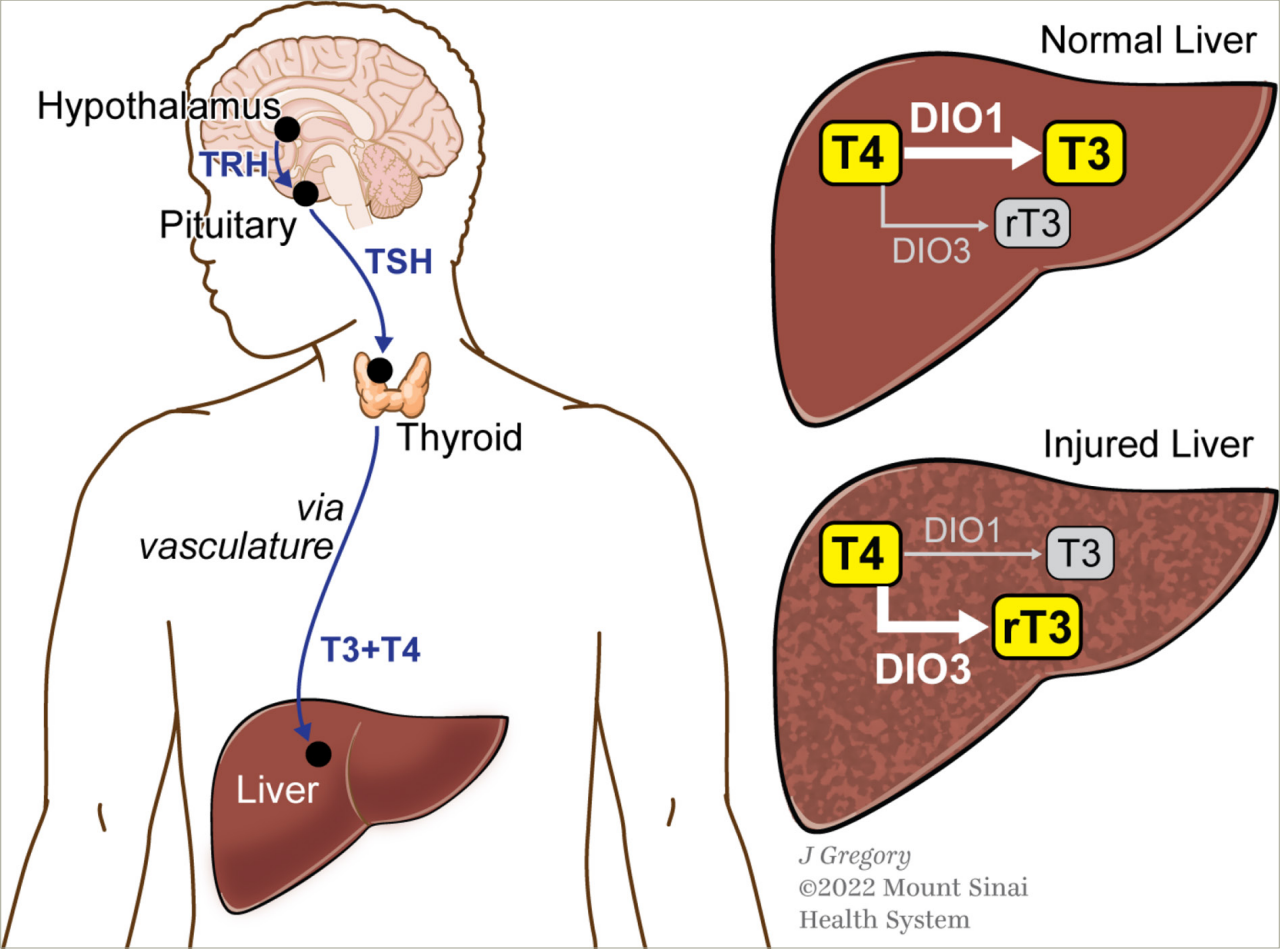
Changes in deiodinase type 1 and deiodinase type 3 in chronic liver injury drives intrahepatic hypothyroidism

*In the normal liver, DIO1 activity, an intrahepatic enzyme, drives the conversion of T4 to active T3. In chronic liver injury, a decrease in DIO1 activity coupled with an increase in DIO3 activity leads to increased conversion of T4 to inactive reverse T3, subsequently leading to accumulation of lipotoxic species, cyclic liver injury and local intrahepatic hypothyroidism. Used with permission from ©Mount Sinai Health System.*

*DIO1 = deiodinase type 1; DIO3 = deiodinase type 3; rT3 = reverse triiodothyronine; T3 = triiodothyronine; T4 = thyroxine; TRH = thyroid-releasing hormone; TSH = thyroid-stimulating hormone.*

Moreover, the decreases in the free T3 to rT3 and free T4 to rT3 ratios distinguished advanced from mild fibrosis, even in individuals with similar serum levels of TSH and free T4.^33^ Therefore, it is thought that with chronic liver injury, intrahepatic thyroid hormone signalling may be impaired; this impairment would decrease conversion of T4 by DIO1 to active T3 and increase conversion of T4 by DIO3 to inactive rT3, leading to the accumulation of lipotoxic species, stimulating a cycle of repetitive liver injury.^33^ Taken together, these findings suggest that intrahepatic hypothyroidism may be a driver of NASH pathogenesis (*Figure 2*).

### Specific role of thyroid hormone receptor-β in hepatic lipid metabolism

**Figure 3:**
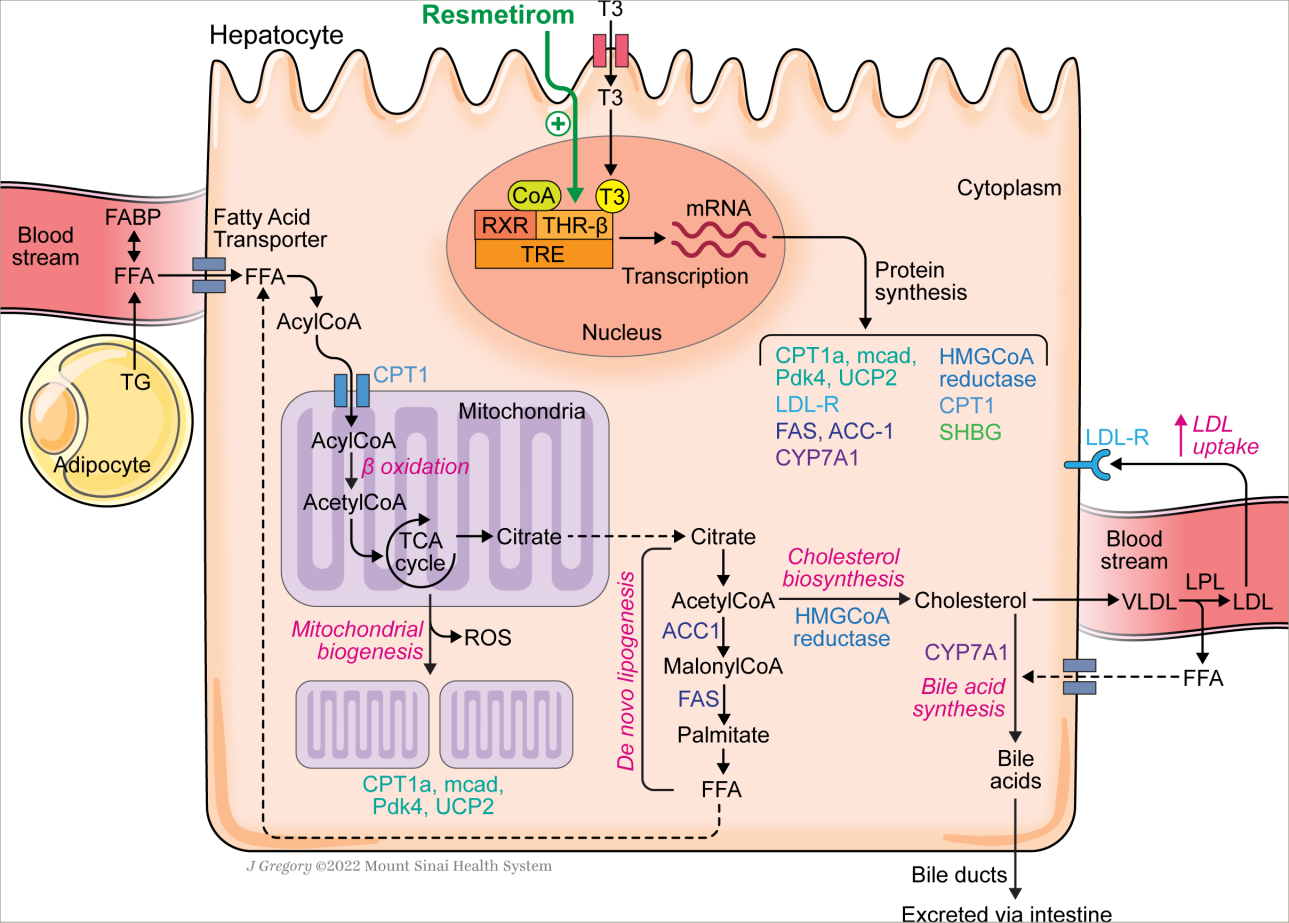
Proposed hepatocyte pathways activated by thyroid hormone receptor-β agonists in relation to non-alcoholic steatohepatitis

Hepatocytes highly express THR-β, which is responsible for regulating metabolic pathways that are impaired in NAFLD and NASH.^34^ Most hepatic fat is derived from free fatty acids released by adipocytes. In NASH, β-oxidation of hepatic lipids is decreased, resulting in lipotoxicity.^35^ Animal studies have shown that the activation of THR-β plays an important role in reducing triglycerides and cholesterol.^33,36^ In cultured HepG2–THR-β cells of a mouse model, thyroid hormone treatment increased mitochondrial respiration and fatty acid oxidation under basal and palmitic acid-treated conditions and decreased lipopolysaccharides and palmitic acid-stimulated inflammatory and fibrotic responses.^32^ *Figure 3* summarizes and highlights some of the hepatocyte pathways thought to be upregulated by THR-β agonists and most relevant to NASH.

In one study, patients with THR-β mutations had increased liver fat (as assessed by controlled attenuation parameters using transient elastography) compared with their unaffected family members, while controlling for body mass index.^37^ All participants belonged to the same family, lived on the same small island and were, therefore, exposed to similar environmental conditions. No difference in insulin resistance was observed between the two groups. Individuals with NASH display low THR-β activity, which exacerbates mitochondrial dysfunction and lipotoxicity.^35^ Given the role played by THR-β signalling in liver metabolism, a strong rationale for developing THR-β-selective thyromimetics exists. Consequently, new molecules with a very high THR-β affinity and hepatic selectivity have been developed to treat lipid-a ssociated hepatic disorders, in particular NAFLD.

### Thyroid hormone receptor-β agonists for the treatment of non-alcoholic fatty liver disease/non-alcoholic steatohepatitis

For the treatment of NASH, a THR-β agonist ideally needs to achieve three goals: 1) reduced hepatic steatosis, inflammation and fibrosis; 2) liver specificity with no effect on the hypothalamus-pituitary-thyroid axis, which regulates serum thyroid levels; 3) high THR-β selectivity to limit off-target THR-α effects on the bone/cartilage and heart.

Sobetirome (GC-1) and eprotirome (KB2115) were the first THR-β agonists shown to reduce intrahepatic lipid content in preclinical models.^38,39^ Despite its favourable lipid-l owering effects, eprotirome development was halted in phase III because it caused cartilage damage in dogs following chronic treatment.^40^ In addition, liver toxicity was noted when patients with familial hypercholesterolaemia were treated after only 6 weeks.^34,40^ Sobetirome had beneficial effects on lipid profile by upregulating low-density lipoprotein (LDL) receptors on hepatocytes, and reduced hepatic steatosis in a rat model of NAFLD. Despite these favourable results, funding limited its advancement to phase I, along with some experimental observations of hyperglycaemia and insulin resistance.^41^ Interestingly, Sob-AM2, a methyl amide derivative of sobetirome, which has greater central nervous system penetration, is being investigated for treating demyelinating diseases in preclinical models.^42^ Novel 4’-amino-benzyl-phenoxyacetic acid thyromimetics IS25 and the prodrug TG68 showed promise in preclinical testing, and additional *in vivo* studies will be needed to determine their potential for treating NAFLD and NASH.^34^ A novel glucagon/T3 hybrid molecule, which is meant to have both the anti-l ipid effects of glucagon along with the positive energy effects of T3, has recently been developed.^43^ Preclinical models using this molecule in obese mice found decreased serum lipids, decreased adipose mass, reversal of NASH, reduced atherosclerotic plaque accumulation and improved glucose metabolism, awhile avoiding thyrotoxicosis and the hyperglycaemic effects of glucagon.^43^ Although additional studies are still needed to advance the molecule, it is notable that this approach does not rely on isoform selectivity to produce its beneficial effects.

*It is hypothesized that THR-β agonists, such as resmetirom, activate THR-β, an abundant nuclear hormone receptor found hepatocytes. It is hypothesized that activating THR-β modulates several genes that promote uptake of free fatty acids from both external sources (CPT1), as well as increased production and uptake of internal free fatty acids from* de novo *lipogenesis (ACC1, FAS) and lipophagy. Increased mitochondrial biogenesis (CPT1a, mcad, PDk4, UCP2) and mitophagy of unhealthy mitochondria increase capacity to handle and burn the increased flow of free fatty acids. While increased cholesterol is produced due to upregulation of HMGCoA reductase, bile acid synthesis increases through increased CYP7A1 and excretion, and LDL uptake by the liver increases by induction of the LDL-R. As a result, fatty acid β-oxidation, mitochondrial biogenesis,* de novo *lipogenesis, cholesterol and bile acid synthesis all increase, and LDL cholesterol levels decrease. SHBG, which transports androgens and oestrogens in blood and regulates their access to target tissues, is also a known downstream target of THR-β and may be a biomarker of target engagement.*

*Used with permission from ©Mount Sinai Health System.*

*ACC1 = acetyl coenzyme A carboxylase 1; acetylCoA = acetyl coenzyme A; acylCoA = acyl coenzyme A; CPT1 = carnitine palmitoyltransferase I; CYP7A1 = cholesterol 7 alphahydroxylase; CoA = coenzyme A; FABP = fatty acid binding protein; FAS = fatty acid synthase; FFA = free fatty acids; HMGCoA = 3-hydroxy-3-methyl-glutaryl-coenzyme A reductase; LDL = low-density lipoprotein; LDL-R = low-density lipoprotein receptor; mcad = medium-chain acyl-coenzyme A dehydrogenase; Pdk4 = pyruvate dehydrogenase kinase 4; ROS = reactive oxygen species; RXR = retinoid X receptor; SHPG = sex hormone binding globulin; T3 = triiodothyronine; TCA = tricarboxylic acid; TG = triglycerides; THR-β = thyroid hormone receptor β; TRE = tetracycline-responsive element; UCP2 = uncoupling protein 2; VLDL = very-low-density lipoprotein.*

The THR-β agonists that are furthest along in development for the treatment of NASH are resmetirom (MGL-3196), VK2809 (MB07811), ASC-41 and TERN 501. VK2809 is a prodrug activated by cytochrome P4503A (CYP3A) in the liver and is being investigated for the treatment of NASH with F2/F3 fibrosis in a phase IIb clinical trial ( ClinicalTrials. gov identifier: NCT04173065),^44^ and TERN-501 is being investigated for the treatment of NASH in a phase IIa (C linicalTrials. gov identifier: NCT05415722).^45^ ASC-4, also a prodrug metabolized by CYP3A, is being investigated in a phase II trial and is limited to China ( ClinicalTrials. gov identifier: NCT05118360).^46^ Completed and on-going trials with other THR-β agonists are summarized in *Table 2*.^47–51^ Data shown for all agents, with the exception of resmetirom, are derived from presentations at international meetings or results presented on company websites and, thus, have not been subject to peer review.

**Table 2:** Thyroid hormone receptor-β agonists in development for non-alcoholic fatty liver disease/non-alcoholic steatohepatitis.

Completed studies with THR-β agonists				
Study	Study design	LDL	Liver fat	LFT
MGL-3196 (phase IIb)^47^	125 patients with biopsy-proven NASH; 36 weeks; 60 or 80 mg daily	LDL-C -11% (placebo +6%)	Liver fat -50% (80 mg) (placebo -14%)	AST, ALT↓
VK-2809 (MB07811) (phase IIa)^48^	42 patients with NAFLD and increased LDL-C; 12 weeks; 5 mg/10 mg every other day or 10 mg daily	LDL-C -18% (placebo +2%)	Liver fat -60% (10 mg) (placebo -9%)	AST, ALT↓
TERN-501 (phase I)^49^	18 patients with elevated LDL-C (not NAFLD); 14 days increasing dose 1 mg to 10 mg	LDL-C -20% (placebo -4%)	N/A	N/A
ASC41 (phase I)^50^	36 patients with LDL-C >110mg/dL (not NAFLD); 14 day once daily oral dosing (1 mg, 2 mg, 5 mg)	5 mg dose placebo-adjusted LDL-C -20%	N/A	N/A
ASC41 (phase I)^51^	20 patients overweight/obese with LDL-C >110 mg/dL (not NAFLD); 10 mg daily for 14 and 28 days	n=13; 14 day placebo-adjusted LDL-C -39%; 28 day placebo-adjusted LDL-C -37%	N/A	N/A
**On-going phase II studies with non-resmetirom THR-β agonists**				
**Study (ClinicalTrials.gov identifier)**	**Phase of development**	**Study population**	**Primary endpoints**	**Key secondary endpoints**
VK-2809; VOYAGE (NCT04173065)	IIb	NASH with F2/F3 fibrosis	Relative change in liver fat content (assessed by MRI-PDFF) from baseline to Week 12 compared with placebo	52 weeks: Proportion of subjects with resolution of steatohepatitis on overall histopathological reading and no worsening of liver fibrosis on NASH CRN fibrosis score
TERN-501; DUET (NCT05415722)	IIa	Presumed NASH based on biopsy or imaging. Monotherapy: TERN 501; Dual therapy: TERN 501+ 101 (FXR agonist)	Relative change from baseline to Week 12 in MRI-PDFF for TERN-501 monotherapy (arms 1, 2 and 3) compared with placebo	1. Change from baseline to 12 weeks in cT1 relaxation time for TERN-501 monotherapy (arms 1, 2 and 3) compared with placebo; 2. Relative change from baseline to 12 weeks in MRI-PDFF for TERN-501+TERN-101 combination (arms 4 and 5) compared with placebo; 3. Change from baseline to 12 weeks in cT1 relaxation time for TERN-501+TERN-101 combination (arms 4 and 5) compared with placebo; 4. Patient incidence of treatment emergent adverse events for all treatment groups (16 weeks)
ASC-1 (NCT05118360)	II	NASH on liver biopsy; NASH F1 (up to 15%), F2, or F3, NAS ≥4	52 weeks: Resolution of NASH with at least 2-point reduction in NAS (improvement in inflammation or ballooning) with no worsening of fibrosis; OR reduction in fibrosis stage by 1 point with no worsening of NAS	LDL-C lowering at Weeks 12, 24 and 52

*Only studies with MGL-3196 (resmetirom) and VK-2809 included patients with NAFLD. All compounds show promising results in their lipid-lowering effects.*

↓ *= decrease; LDL = low-density lipoprotein; LDL-C = low-density lipoprotein-cholesterol ; LFT = liver function tests; MRI-PDFF = magnetic resonance imaging proton density fat fraction; N/A = not available; NAFLD = non-alcoholic fatty liver disease; NAS = non-alcoholic fatty liver disease score; NASH = non-alcoholic steatohepatitis.*

## Summary of preclinical data for resmetirom

Resmetirom is an investigational liver-directed agonist of THR that is 28 times more selective for THR-β over THR-α.^52^ *In vitro* studies have demonstrated resmetirom’s preferential use of the organic anion transporting polypeptides 1B1 receptor for hepatocyte uptake and stronger activation of THR-β.^53,54^ Moreover, oxygen consumption rate studies found that both T3 and resmetirom increased basal and maximal respiration, accompanied by an increase in the production of adenosine triphosphate, reflecting higher oxidation of substrates.^53^ While it is known that increased hepatic THR-β agonism increases the level of hepatic DIO1,^55,56^ additional *in vitro* and *in vivo* data suggest that resmetirom upregulates the expression of DIO1, supporting its role not only as a thyromimetic but also in potentially increasing the conversion of T4 to T3.^54^ Although not definitive, the low serum T4 detected in patients treated with resmetirom indirectly suggests this mechanism may be relevant.^53^

Given its specificity for THR-β and its impact on lipophagy, mitophagy, mitogenesis and β-oxidation within hepatocytes, resmetirom is an appealing agent for treating NASH.

## Summary of completed clinical trial data

The first-i n-human data on resmetirom in healthy volunteers came from a single-centre, randomized, double-blind, placebo-controlled, ascendingdose study in 72 subjects to assess its safety and pharmacokinetics ( ClinicalTrials. gov identifier: NCT01367873) and a 2-week multiple-dose study (5–200 mg) in 48 healthy participants with mildly elevated LDL (>110 mg/dL) to assess its safety, pharmacokinetics, impact on thyroid axis hormones and LDL cholesterol (LDL-C) lowering effects ( ClinicalTrials. gov identifier: NCT01519531).^57^ Resmetirom was tolerated well at all doses, and no dose-related adverse events or changes in electrocardiogram findings, vital signs or liver enzyme levels were reported. Compared with placebo, patients receiving resmetirom displayed a reduction of up to 30% in LDL-C, 28% in non-high-density lipoprotein-cholesterol, 24% for in apolipoprotein B and 60% in triglycerides compared with those receiving placebo. At the highest dose (200 mg), there was a significant (p<0.0001) reversible reduction of approximately 20% in the level of prohormone free T4 compared with placebo;^57^ this could theoretically be explained by increased hepatic metabolism of T4 by DIO1, but has not yet been proven.^55^ No impact on the central thyroid axis was observed, as reflected by the lack of change in TSH or free T3 levels. In conclusion, in this 2-week study, resmetirom was well tolerated and demonstrated favourable effects on lipid metabolism in healthy volunteers with mild elevation in LDL-C.^57^ Given that cardiovascular disease is the leading cause of death in patients with NASH, the favourable cardiometabolic profile of resmetirom and the preclinical data for intrahepatic thyroidism as a driver of lipotoxicity in NASH, resmetirom was advanced to phase II/ III trials in patients with NASH.

The first seminal clinical trial was a 36-week, phase II, serial liver biopsy study ( ClinicalTrials. gov identifier: NCT02912260) in patients with biopsyconfirmed NASH (fibrosis stages 1–3), with a NAFLD activity score (NAS) ≥4 and hepatic fat fraction of ≥10% at baseline when assessed by MRI-PDFF.^47^ The NAS comprises three components: steatosis (0–3), ballooning (0–2) and inflammation (0–3). The maximum NAS is 8, and patients had to have a score ≥4 with the requirement of ballooning for inclusion.

**Figure 4.**
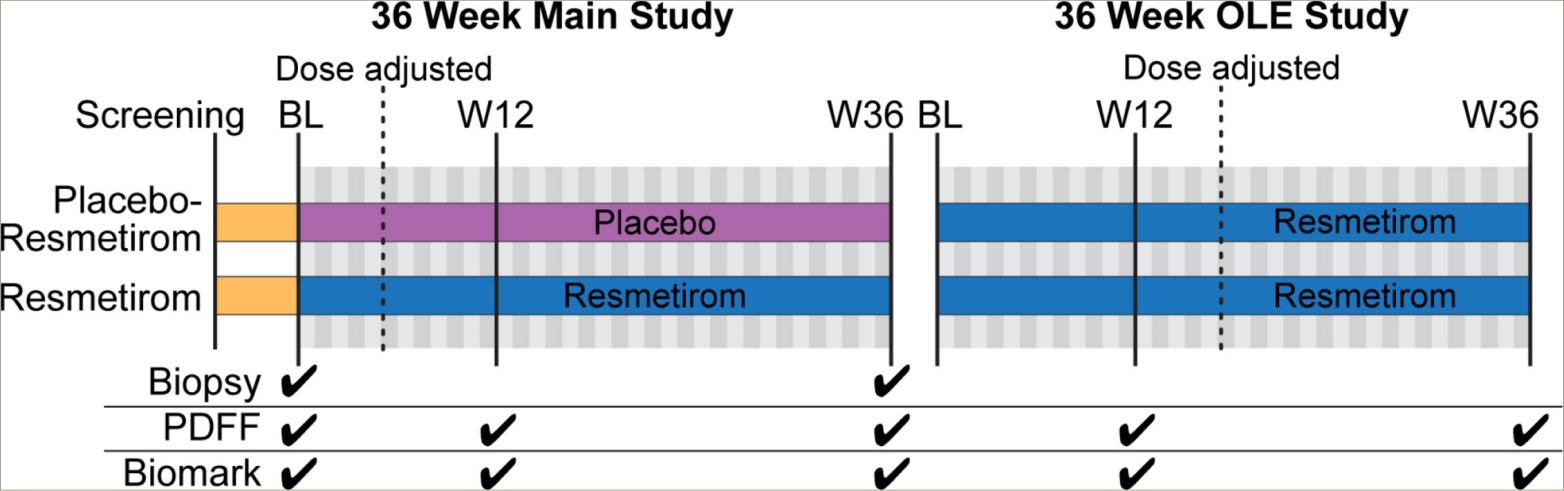
Treatment schematic and design of phase II MGL-3196-05 (ClinicalTrials.gov identifier: NCT02912260) 36-week main and open-label extension studies Used with permission from ©Mount Sinai Health System.

Patients were randomly assigned to receive either resmetirom 80 mg or placebo orally once a day. Serial hepatic fat measurements were obtained at Weeks 12 and 36, and a second liver biopsy was obtained at Week 36 (*Figure 4*; the 36-week main study).^47^ The primary goal of this study was to determine whether resmetirom could effectively reduce the burden of lipotoxic lipids that may be driving liver injury. Therefore, the primary endpoint was a relative change in MRI-P DFF fat quantification at 12 weeks in patients on resmetirom compared with placebo. Key secondary endpoints included the proportion of patients who had achieved a reduction in hepatic fat of ≥30% at 12 and 36 weeks on MRI-PDFF, and critical liver biopsy assessments evaluating significant improvements in NASH and fibrosis as dictated by the FDA. Reduction in ballooning and inflammation are critical, as a simple reduction in steatosis in isolation could result in a 2-point reduction in NAS without any impact on the more relevant features of ballooning and inflammation.

At Week 12 of treatment, patients treated with resmetirom (n=78) demonstrated both a relative reduction (-32.9% versus -10.4%) and an absolute reduction (-7.0% versus -2.7%) of hepatic fat compared with patients on placebo (n=38; p<0.0001). At Week 36, patients treated with resmetirom (n=74) showed both a statistically significant relative (-37.3% versus -8.9%) and absolute (-8.2% versus -2.8%) reduction in hepatic fat compared with patients on placebo (n=34; p<0.0001) (*Table 3*).^47^ Furthermore, resmetirom seemed to produce statistically significant reductions in a number of secondary and exploratory endpoints, including liver enzymes, multiple lipoproteins and atherogenic lipids (*Table 4*).^47^ rT3 levels were also significantly reduced in resmetirom-treated patients, suggesting an impact on intrahepatic metabolism of T4 towards active T3, possibly due to increased DIO1 activity. Resmetirom exposure was assessed by Week 2 through pharmacokinetic studies, and dose adjustments were made at Week 4 by an unblinded monitor. As sex hormone binding globulin (SHBG) is a known downstream target of THR-β agonism in the liver, it was assessed as a potential surrogate marker of target engagement and hepatic exposure to resmetirom, with a high SHBG group defined as a ≥75% and ≥88% increase in SHBG concentration from baseline to Week 12 and 36, respectively. Therefore, secondary and exploratory endpoints were broken down by exposure level and SHBG response.

Although there was not a significant difference in the number of patients achieving ≥1 stage improvement in fibrosis on resmetirom compared with placebo at the Week 36 liver biopsy, several key observations were made in assessing the impact of resmetirom on NASH and predictors of response. Specifically, the proportion of patients with a 2-point reduction in NAS and at least a 1-point reduction in either ballooning or inflammation at the Week 36 biopsy was significantly greater in the resmetirom group compared with the placebo group but only in certain subgroups of patients, including those who had <5% weight loss (p=0.017), those with high resmetirom exposure (p=0.021) and those who were MRI-PDFF responders by Week 12 (p=0063). This last finding suggests that early reduction in hepatic fat could predict who is likely to have a response by the Week 36 liver biopsy. Indeed, of the 46 patients in the resmetirom group who were MRI-PDFF responders at Week 12, 18 (39%) achieved NASH resolution (p=0.0013), defined as a ballooning score of 0, inflammation of 0–1 with a ≥2 point reduction in NAS score. Change in absolute NAS was not significantly different between groups but did show a difference in those with high exposure, high SHBG and Week 12 MRI-PDFF response (*Table 5*).^47^ The authors suggested that thyroid hormone-responsive protein SHBG may serve as a biomarker for monitoring compliance and optimizing dosing. There was no difference in patients who achieved NASH resolution with no worsening of fibrosis, except in a subgroup analysis of those with <9.5% weight loss and MRI-PDFF response, defined as >30% reduction of MRI-PDFF at Week 12.

*BL = baseline; OLE = open-label extension; PDFF = proton density fat fraction; W12 = week 12; W36 = week 36.*

**Table 3:** Changes in hepatic fat content in the phase II MGL-3196-05, 36 week main study^1^

	Resmetirom 80 mg, % (SE)	Placebo, % (SE)	Least-squares difference from baseline, % (95% Cl)	Odds ratio for NASH resolution (95% Cl)	p-value
Relative change in MRI-PDFF from baseline to Week 12,	-32.9 (3.0)	-10.4 (4.3)	-22.5 (-32.9,-12.2)	N/A	<0.0001
Relative change in MRI-PDFF from baseline to Week 36	-37.3 (3.7)	-8.9 (5.4)	-28.4 (-41.3,-15.4)	N/A	<0.0001
Absolute change in MRI-PDFF from baseline to Week 12	-7.0 (0.6)	-2.7 (0.8)	-4.3 (-6.3, -2.4)	N/A	<0.0001
Absolute change in MRI-PDFF from baseline to Week 36	-8.2 (0.7)	-2.8 (1.1)	-5.3 (-7.8, -2.8)	N/A	<0.001
Liver biopsy at Week 36, change in NAS	-1.4 (0.14)*	-1.0 (0.21)*	-0.4 (-0.9, 0.1)	N/A	0.082
Patients with >30% fat reduction at Week 12	60.30^+^	18.40^+^	N/A	6.8 (2.6-17.6)	<0.0001
Patients with >30% fat reduction at Week 36	67.60^+^	29.40^+^	N/A	4.9 (2.0-11.9)	0.0006

*At both week 12 of treatment (primary endpoint) and week 36, resmetirom-treated patients (n=78) demonstrated both a relative and an absolute reduction of hepatic fat as assessed by MRI-PDFF when compared with placebo (n=38). Those that had a >30% reduction in hepatic fat at 12 and 36 weeks had an odds ratio of 6.8 and 4.9, respectively for achieving NASH resolution.*

*Values are mean (SE).

*^f^Values are %.*

*Cl = confidence interval; MRI-PDFF = magnetic resonance imaging proton density fat fraction; N/A = not applicable; NAS = non-alcoholic fatty liver disease score; NASH = non-alcoholic steatohepatitis; SE = standard error.*

**Table 4:** Change in lipids and liver enzymes in the phase II MGL-3196-05, 36-week main study^47^

	Placebo, % mean (SE)	Resmetirom, % mean (SE)	Least-squares mean difference, % (95% CI)	p-value
LDL-C, mg/dL	6.2 (3.1)	-11.2% (2.1)	-17.3% (-24.8, -9.9)	<0.0001
HDL-C, mg/dL	2.2 (3.4)	6.0% (2.3)	3.8% (-4.4, 12.0)	0.36
Lp(a) (baseline >10 nmol/L), nmol/L	15.3% (8.9)	-22.7% (6.3)	-37.9% (-59.7, -16.2)	0.0009
Apolipoprotein B (baseline LDL-C ≥100 mg/dL), mg/dL	7.4% (3.5)	-20.2% (2.5)	-27.6% (-36, -19.1)	<0.0001
Triglycerides, mg/dL	20.5% (5.5)	-15.4% (3.8)	-36.0% (-49.2, -22.7)	<0.0001
Apolipoprotein CIII, mg/dL	24.5% (5.4)	-12.0% (3.7)	-36.5% (-49.6, -23.5)	<0.0001
Alanine aminotransferase, IU/L				
Week 12 Week 36	-5.2 (3.9) 11.0 (6.8)	-8.2 (2.7) -15.4 (4.7)	-3.0 (-12.4, 6.4) -26.4 (-42.8, -9.9)	0.53 0.0019
Aspartate aminotransferase, IU/L				
Week 12 Week 36	-1.1 (2.5) 3.6 (2.8)	-5.8 (1.8) -7.4 (1.9)	-4.8 (-10.9, 1.4) -11.1 (-17.8, -4.3)	0.13 0.0016
Gamma-glutamyl transpeptidase, IU/L	49.4 (15.2)	-9.1 (10.4)	-58.5 (-95.2, -21.8)	0.002
Reverse triiodothyronine, ng/dL	-1.37 (0.73)	-4.26 (0.50)	-2.88 (-4.64, -1.12)	<0.0001

*Resmetirom therapy was associated with a statistically significant reduction in atherogenic lipids and liver enzymes at Week 36. Resmetirom significantly decreased serum reverse triiodothyronine.*

*CI = confidence interval; HDL-C = high-density lipoprotein-cholesterol; LDL-C = low-density lipoprotein-cholesterol; Lp(a) = Lipoprotein (a); SE = standard error.*

Overall, this study met its primary endpoint of MRI-PDFF reduction at Week 12. Moreover, resmetirom responders with a 30% MRI-PDFF reduction at Week 12 had higher rates of NASH resolution (37%) at the Week 36 liver biopsy compared with non-responders (4%) along with a reduction in liver enzymes and fibrosis biomarkers, suggesting that early MRI-PDFF response may predict future NASH resolution and anti-fibrotic effect. In patients treated with resmetirom, a higher incidence of mild diarrhoea and nausea was reported; however, most adverse events were mild, selflimiting and balanced between the two groups studied. There were no reports of adverse events related to THR-α activity, such as changes in bone mineral density based on dual-energy X-ray absorptiometry (DEXA) scan, thyroid axis suppression or cardiovascular adverse events.^47^

At the end of the main 36-week, phase II study, a 36-week, activetreatment, open-l abel extension (OLE) study was conducted in 31 patients with persistently mild to markedly elevated liver enzymes (*Figure 4*).^58^ All OLE study endpoints were exploratory. The main efficacy outcome was absolute and relative reductions in MRI-PDFF at Week 36. At Week 36, patients treated with resmetirom 80 mg or 100 mg demonstrated a mean absolute reduction in MRI-PDFF of -11.1% (standard error 1.5%; p<0.0001) and mean relative reduction of -52.3% (standard error 4.4%, p<0.0001). Interestingly, the controlled attenuation parameter score, which is commonly used to assess steatosis with FibroScan® (Echosens, Paris, France), did not correlate with changes in MRI-PDFF, suggesting that it may not be a reliable assessment of steatosis reduction in response to treatment. Other key outcomes, including liver enzymes, lipid profiles and markers of fibrogenesis, were favourably reduced and are shown in *Table 6*.^58^ While no repeat liver biopsy was conducted at the end of the OLE study, several markers of fibrogenesis were assessed. Of particular interest were PRO-C3 and C3M levels, which had not been initially analysed in the main 36-week study. PRO-C3 reflects the production of the pro-peptide (N-terminal pro-collagen) of type III collagen and is thought to be a biomarker of new collagen production by activated stellate cells or fibrogenesis. On the other hand, C3M is the matrix metallopeptidase 9-mediated degradation product of type III collagen and, thus, reflects fibrinolysis. Therefore, the PRO-C3/C3M ratio is thought to reflect net fibrosis production.^59^ In the *post hoc* analysis from the main study, the PRO-C3/C3M ratio significantly correlated with fibrosis stage (p=0.001) and ballooning (p=0.003) on liver biopsy. It did not correlate with other NAS components.^58^ During the OLE study, similar to the main study, PRO-C3, a neo-epitope pro-peptide of type III collagen formation, was reduced significantly with resmetirom treatment (p=0.0005), and C3M increased such that the PRO-C3/C3M ratio decreased significantly with treatment (p<0.0001).^58^ Consistent with these changes, liver stiffness calculated using vibration-controlled transient elastography on FibroScan® showed statistically significant (p=0.015) improvement during the 36-week OLE study and may serve as a convenient point-of-care assessment for treatment effects in a real-world setting.^60^ In terms of impact on the thyroid axis, treatment with resmetirom significantly reduced serum rT3 and increased the free T3/rT3 ratio. The extent to which this reflects a correction in hepatic thyroid activity is not clear, but is postulated. While no effect on TSH was observed, free T3 did increase by 4%, which requires further monitoring in a longer-term follow-up. Overall, results from the 36-week phase II study and the 36-week OLE study demonstrated safety and efficacy, informed dosing, and suggested the potential role of non-i nvasive assessments in evaluating response to resmetirom, further influencing use in the phase III NASH study MAESTRO-NASH ( ClinicalTrials. gov identifier: NCT03900429).^47,58,61^ Interestingly, using data from the main study, those with a ≥30% reduction in fat on MRI-PDFF by Week 12 were independently associated with greater improvements in physical functioning and Physical Component Summary scores at Week 36 (p<o.05).^62^ Patients with improvement in NASH and fibrosis on liver biopsy also showed improvement in components of health-related quality of life.^47^ Whether this improvement is seen in longer-term studies remains to be evaluated, but these results are encouraging as the regulatory agencies want to ensure that any therapy makes a patient function and feel better.

**Table 5:** Results of the liver biopsy from the 36-week MGL-3196-05 study

Change in NAS at liver biopsy	n	Placebo	n	Resmetirom	Least square mean difference (95% CI)	p-value
Overall, change in NAS mean (SE)	34	-1.0 (0.21)	73	-1.4 (0.14)	-0.4 (0.9–0.1)	0.082
High exposure group	-		43	-1.6 (0.18)	-0.6 (-1.2, 0.1)	0.029
Low exposure group			30	-1.2 (0.22)	-0.2 (-0.8, 0.4)	0.51
High SHBG group			44	-1.7 (0.18)	-0.7 (-1.2, -0.1)	0.016
Low SHBG group			29	-1.1 (0.22)	-0.1 (-0.7, 0.5)	0.77
MRI-PDFF responders by Week 12 (>30% reduction)			46	-1.9 (0.16)	-0.9 (-1.4 to 0.4)	0.0006

*Subgroup analyses identified factors associated with greater histologic response. No significant difference in overall NAS was seen with resmetirom vs placebo. In those with high drug exposure compared with low exposure ( assessed by 24 h area under the curve), high SHBG levels (defined as an 88% increase from baseline), compared with low SHBG, and those who had a ≥30% fat reduction (assessed by MRI-PDFF) had a statistically significant decrease in NAS.*

*MRI-PDFF = magnetic resonance imaging proton density fat fraction; NAS = non-alcoholic fatty liver disease score; SHBG = sex hormone binding globulin.*

**Table 6:** Change from baseline in lipids, liver enzymes, liver stiffness (vibration-controlled transient elastography) and fibrogenesis biomarkers in patients taking resmetirom in a 36-week, open-label extension, phase II study (ClinicalTrials. gov identifier: NCT02912260)^58^

	Mean change from baseline (SE)	p-value
LDL-C*, mg/dL	-39.8 (8.4)	<0.0001
HDL-C*, mg/dL	-1.7 (1.2)	0.72
Apolipoprotein B* (baseline LDL-C≥100 mg/dL), mg/dL	-32.8 (6.6)	<0.0001
Triglycerides* (baseline ≥150 mg/dL), mg/dL	-70.8 (21.7)	0.0016
Apolipoprotein CIII, mg/dL	-2.8 (0.62)	<0.0001
ALT, IU/L	-23.3 (6.7)	0.0016
AST, IU/L	-8.1 (4.1)	0.061
GGT, IU/L	-24.1 (6.2)	0.0006
PRO-C3, ng/mL	-7.32 (1.9)	0.0005
C3M, ng/mL	-0.54 (0.37)	<0.0001
PRO-C3/C3M	-0.68 (0.15)	<0.0001
FibroScan® VCTE, kPa	-2.1 (0.8)	0.015
FibroScan® CAP	-12.4 (11.3)	0.29
SHBG, nmol/L	74.9 (10.1)	<0.0001

*The open-label extension found that patients taking resmetirom displayed statistically significant reductions in atherogenic lipids, ALT, GGT, PRO-C3/C3M and liver stiffness (measured using VCTE) compared with baseline. On the other hand, no difference was observed for AST levels and CAP score, while increased SHBG levels reflected target engagement.*

All parameters were assessed at Week 36, except those with * which were assessed at Week 32.

*Assessed at Week 32.

*ALT = alanine transaminase; AST = aspartate aminotransferase; CAP = controlled attenuation parameter; GGT = gamma-glutamyl transpeptidase; HDL-C = high-density lipoprotein-cholesterol; LDL-C = low-density lipoprotein-cholesterol; SE = standard error; SHBG = Sex Hormone Binding Globulin; VCTE = vibration-controlled transient elastography.*

### Positive phase III results for non-thyroid hormone receptor-β agonists in the treatment of non-alcoholic steatohepatitis: obeticholic acid

While the focus of this review is on resmetirom, obeticholic acid (OCA) has also demonstrated positive phase III results for NASH. OCA is a bile acid analogue that works by activating the farnesoid X receptor and received FDA approval for the treatment of patients with primary biliary cholangitis in 2016.^63^ In a large, multicentre, phase IIb clinical trial, patients who received OCA (25 mg/day) demonstrated improved steatohepatitis and fibrosis over 72 weeks.^64^ In the phase III clinical trial, OCA significantly improved fibrosis and NASH disease activity among patients with NASH.^65^ Due to its mechanism of action, OCA inhibits the synthesis of bile acids from cholesterol and thus results in a mild increase in cholesterol and the incidence of gallstones in patients on therapy. Pruritis is a common side effect that can cause therapy discontinuations in about 10% of patients. An on-going phase III study is assessing the long-term efficacy and safety of OCA ( ClinicalTrials. gov identifier: NCT02548351) and is under review by the FDA.^66^ It is possible that OCA will be the first approved therapy for NASH, with submission of resmetirom data to the FDA to follow.

### Future directions and anticipated focus of new clinical trials

Based on its safety, tolerability and efficacy as reflected by non-i nvasive assessments, resmetirom has established itself as a leader in the crowded field of potential NASH therapeutics. While not yet published and thus not discussed extensively, 52-week serial biopsy data including 950 patients from the MAESTRO-NASH trial were released publicly in top-l ine clinical results at the end of 2022.^67^ Both primary outcomes were met with 1) NASH resolution (ballooning of 0, inflammation of 0–1) and ≥2-point NAS reduction, with no worsening of fibrosis (p<0.0001 at both doses), and 2) fibrosis improvement by at least one stage, with no worsening of NAS (p=0.0002 and p<0.0001 at 80 mg and 100 mg, respectively). We await a formal peer-reviewed publication and Subpart H (Accelerated Approval of New Drugs for Serious or Life-Threatening Illnesses) filing with the FDA. While the MAESTRO-NAFLD-OLE study focuses on the safety and tolerability of an additional 52 weeks of resmetirom exposure, the extensive secondary and exploratory endpoints provided invaluable information on the monitoring of drug response using various non-invasive assessments of steatosis and more importantly fibrosis.^58^

Thus far, no clinical trials have demonstrated efficacy of resmetirom in a cirrhotic population, largely because they have focused on reducing fibrosis by one stage on liver biopsy as a primary endpoint, or have enrolled patients with too advanced a disease. For a cirrhosis trial, clinical endpoints represent the most critical assessment of efficacy. The MAESTRO-NASH-OUTCOMES trial ( ClinicalTrials. gov identifier NCT05500222) will be enrolling patients with early Child-Pugh class A cirrhosis (score of 5–6) and, through a rigorous adjudication process, define clinical events with a composite clinical endpoint that includes hepatic decompensation events, Model for End-stage Liver Disease score ≥15, liver transplant and all-cause mortality.^68^ Ultimately, it is improvement in clinical outcomes that will move both the FDA and payors.

The path to NASH therapeutics reaching the market has been challenging. Early optimism was tempered by numerous failed trials. However, optimism has been renewed with the promising data collected for resmetirom. Its safety profile, coupled with potential efficacy that will be determined in upcoming data from phase III trials, positions it as a leader in a crowded field of candidate therapeutics.
